# Ion Gel-Modulated Low-Temperature Field-Effect Phototransistors with Multispectral Responsivity for Artificial Synapses

**DOI:** 10.3390/s25092750

**Published:** 2025-04-26

**Authors:** Junjian Zhao, Yufei Zhang, Di Guo, Junyi Zhai

**Affiliations:** 1 Beijing Key Laboratory of Micro-Nano Energy and Sensor, Center for High-Entropy Energy and Systems, Beijing Institute of Nanoenergy and Nanosystems, Chinese Academy of Sciences, Beijing 101400, China; zhaojunjian@binn.cas.cn (J.Z.); zhangyufei@binn.cas.cn (Y.Z.); 2 School of Nanoscience and Engineering, University of Chinese Academy of Sciences, Beijing 100049, China; 3 Center on Nanoenergy Research, School of Physical Science & Technology, Guangxi University, Nanning 530004, China

**Keywords:** ion gel, ink-jet printing, coplanar gating, electric double layer, a-IGZO TFT transistor, artificial synapse

## Abstract

We report an ion-gel-gated amorphous indium gallium zinc oxide (a-IGZO) optoelectronic neuromorphic transistors capable of synaptic emulation in both photoelectric dual modes. The ion-gel dielectric in the coplanar-structured transistor, fabricated via ink-jet printing, exhibits excellent double-layer capacitance (>1 μF/cm^2^) and supports low-voltage operation through lateral gate coupling. The integration of ink-jet printing technology enables scalable and large-area fabrication, highlighting its industrial feasibility. Electrical stimulation-induced artificial synaptic behaviors were successfully demonstrated through ion migration in the gel matrix. Through a simple and controllable oxygen vacancy engineering process involving low-temperature oxygen-free growth and post-annealing process, a sufficient density of stable subgap states was generated in IGZO, extending its responsivity spectrum to the visible-red region and enabling wavelength-discriminative photoresponses to 450/532/638 nm visible light. Notably, the subgap states exhibited unique interaction dynamics with low-energy photons in optically triggered pulse responses. Critical synaptic functionalities—including short-term plasticity (STP), long-term plasticity (LTP), and paired-pulse facilitation (PPF)—were successfully simulated under both optical and electrical stimulations. The device achieves low energy consumption while maintaining compatibility with flexible substrates through low-temperature processing (≤150 °C). This study establishes a scalable platform for multimodal neuromorphic systems utilizing printed iontronic architectures.

## 1. Introduction

The synaptic behavior in the human neural network, characterized by its intricate signal transmission, processing, and memory functions, has long been a cornerstone of biological intelligence. Recent advances in neuromorphic engineering have focused on emulating these biological synapses using photoelectric devices at the hardware level [1,2,3], aiming to develop energy-efficient and scalable artificial neural networks [4,5]. The human brain comprises approximately 10^12^ neurons interconnected by ~10^15^ synapses [1], which underpin complex cognitive processes, including perception, memory formation, and learning behaviors [6]. Therefore, fabricating excitatory or inhibitory synapses in a single device to replicate synaptic computation and memory is a pivotal step toward advanced artificial intelligence (AI) hardware [7].

Electrolyte-gated thin-film transistors (TETs) with electrical double layer (EDL) gate dielectrics have emerged as promising candidates for synaptic devices due to their high specific capacitance (>1 μF/cm^2^) [8], low operating voltage [9], and simplified coplanar structure [8,10]. The EDL’s nanoscale thinness allows for high charge storage density at the electrolyte/semiconductor interface, enabling the low-power modulation of carrier density and mobility in the channel layer [9]. However, solid-state EDL electrolytes pose challenges for flexible substrate integration, while liquid electrolytes lack stability for large-scale device fabrication [11]. Ion gels (IGs), derivatives of ionic liquids (ILs) [12], represent a breakthrough in this field. ILs, composed of asymmetric organic cations and anions, offer high ionic conductivity [13], thermal stability [14], and compatibility with printing techniques [8]. By blending ILs with structuring polymers, IGs retain the high performance of ILs while providing the mechanical stability of solid films [15], enabling scalable [16] processing. Ink-jet (IJ) printing of IGs has been demonstrated to facilitate the low-voltage operation of TETs [17,18], making them ideal for neuromorphic applications. Recent studies have shown that IGs can achieve ultra-high specific capacitances exceeding sub-millisecond polarization response times, critical for synaptic device performance [19].

Despite these advancements, most synaptic devices remain electrically modulated. Expanding this to non-electrical signals, such as light, is crucial given that ~80% of human sensory input is visual [20]. Optoelectronic synaptic transistors, which integrate light-sensitive channel materials and electronic signal processing, hold promise for high-speed parallel computation to facilitate direct optical signal processing [21]. Amorphous indium gallium zinc oxide (a-IGZO), as one of the most extensively investigated transparent amorphous oxide semiconductors (AOSs), exhibits exceptional electronic properties, including high carrier mobility and operational stability, coupled with an intrinsic persistent photoconductivity (PPC) effect [22]. The PPC phenomenon manifests as a pronounced increase in channel conductivity upon photoexcitation, followed by characteristic temporal decay dynamics after light cessation, rather than immediate recovery to baseline conductivity. This behavior, ubiquitously observed in AOSs materials, plays a pivotal role in enabling critical functionalities in neuromorphic phototransistors through light-stimulated charge trapping and detrapping mechanisms [23,24,25]. However, the ~3.0 eV band gap of a-IGZO limits its optoelectronic applications to the near-UV/blue region [26], necessitating strategies to enhance visible light sensitivity. Extensive research has demonstrated that a-IGZO thin films fabricated in pure argon atmospheres exhibit elevated oxygen vacancy concentrations within their bandgap. These defects, particularly subgap states induced by oxygen vacancies, substantially enhance their photoelectric performance by modulating carrier transport and photogenerated charge dynamics [27,28,29]. In 2023, Wan et al. achieved tunable visible light responsivity in IGZO films by precisely regulating the oxygen/argon gas ratio during magnetron sputtering. Leveraging the pronounced conductivity differences that across wavelengths (360 nm, 405 nm, and 532 nm), the engineered IGZO thin films were utilized as photoresistors in artificial visual photoreceptors to mimic the trichromatic selectivity of human cone cells, offering a straightforward yet effective strategy to expand the spectral response range of IGZO-based optoelectronic devices [30].

In this work, we report the development of an a-IGZO-based optoelectronic neuromorphic transistor fabricated on a transparent electronic-grade glass substrate. By optimizing the deposition and processing conditions (150 °C, pure argon atmosphere), stable subgap states were introduced into the a-IGZO layer, extending its photoresponse spectrum to the red light region and enabling wavelength-discriminative synaptic responses to 405 nm, 532 nm and 638 nm visible light pulses. The device architecture features coplanar source/drain and gate indium tin oxide (ITO) electrodes, with a spin-coated photoresist layer pattern, to load the ion gel prepared by ink-jet printing. This design leverages the lateral electric double-layer coupling effect of the ion gel, allowing for low-voltage operation and efficient modulation of the a-IGZO channel. By exploiting ion migration dynamics within the gel, synaptic behaviors under electrical stimulation were successfully emulated. During synaptic behavior simulations involving variable-intensity and wavelength-selective light pulses, the subgap states in IGZO acted as intermediate energy levels interacting with low-energy photons through a multi-step absorption–excitation mechanism, exhibiting distinct characteristics compared to direct inter-band transitions. Utilizing this device, fundamental synaptic functionalities—including short-term plasticity (STP), long-term plasticity (LTP), and paired-pulse facilitation (PPF)—were successfully simulated under both electrical and visible light stimulations. The minimum power consumption for individual electronic synaptic events and photonic synaptic events achieves 4.5 fJ and 17.5 fJ, respectively, demonstrating ultra-low energy operation comparable to that of biological synapse. These results highlight the potential of this optoelectronic synapse device for scalable, flexible platforms implementing printed iontronic architectures toward multifunctional artificial intelligence (AI) neural systems.

## 2. Results and Discussion

**Figure 1:**
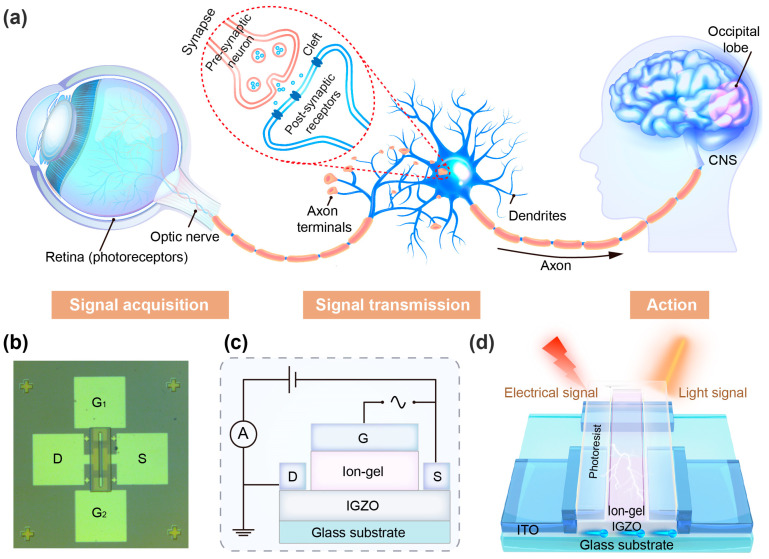
(**a**) A schematic of the human ocular neural system. (**b**) A photograph of the fabricated ion gel-modulated a-IGZO optoelectronic synaptic transistor. (**c**) A schematic illustration of the ion gel-modulated coplanar-gate thin-film transistor structure. (**d**) The operational mechanism of the device under modulation by optical or electrical stimuli.

The visual nervous system, characterized by its intricate architecture and precise functional partitioning, is schematically illustrated in Figure 1a, highlighting the sophisticated interplay between neural components. The human eye serves as optical receptors that can selectively distinguish the three primary colors of light (red, green, and blue) via cone cells in the retina, through specialized photoreceptor mechanisms that converts this physical light stimulus into neural signals, which undergo multiple levels of electrochemical signaling and hierarchical processing within interconnected neural pathways. Such sophisticated integration culminates in the generation of visual perception, ultimately enabling the conscious experience of rich multiscale visual representations [4,5,30,31]. In the formation of vision, light and electrical signals are the main types of signaling mechanisms in biological synapses [5]. Optoelectronic artificial synapses enabling integrated optoelectrical signal integration in monolithic architectures demonstrate superior potential for direct real-time ocular data processing with energy-efficient operation [32]. The ability to bridge the photonic–electronic technology gap is crucial for advancing artificial visual perception-learning systems through emulating biomimetic synaptic plasticity.

Figure 1b provides the micrograph of the fabricated optoelectronic transistor, with detailed fabrication protocols provided in the Materials and Methods section. The device architecture utilizes an electronic-grade transparent glass substrate supporting radio frequency (RF) magnetron-sputtered amorphous indium gallium zinc oxide (a-IGZO) channels. The semiconductor layer was patterned via standard photolithography followed by HCl-based wet etching, with a low thermal budget of 150 °C throughout deposition and processing. Atomic force microscopy (AFM) characterization (Figure S1) reveals sub-nanometer surface roughness (20 × 20 µm^2^, Ra < 0.1 nm), ensuring optimal interfacial contact with the ion gel gate dielectric.

Transparent indium-tin-oxide (ITO) electrodes deposited by RF sputtering at room temperature function as source/drain contacts and lateral gate electrodes, defining channel dimensions of L = 100 μm and W = 500 μm. A critical innovation involves ink-jet-printed ionic liquid patterning (Figure S2) onto the a-IGZO channel and lateral gate, which is subsequently UV-cured into an 1100 × 90 μm^2^ ion gel bridge. This additive manufacturing approach enables scalable fabrication while maintaining photoresist-defined geometries to physically isolate source/drain electrodes from the ionic pathway preventing parasitic ionic conduction. The transistor circuit governed by double-layer (EDL) capacitance modulation at the gate/channel interface is schematically illustrated in Figure 1c. Notably, capacitance-frequency measurements reveal exceptionally high EDL capacitance (2.78 μF/cm^2^), essential for strong gate field coupling. Figure 1d delineates the operation of synaptic emulation, where optical and electrical stimuli, respectively modulate carrier dynamics.

**Figure 2:**
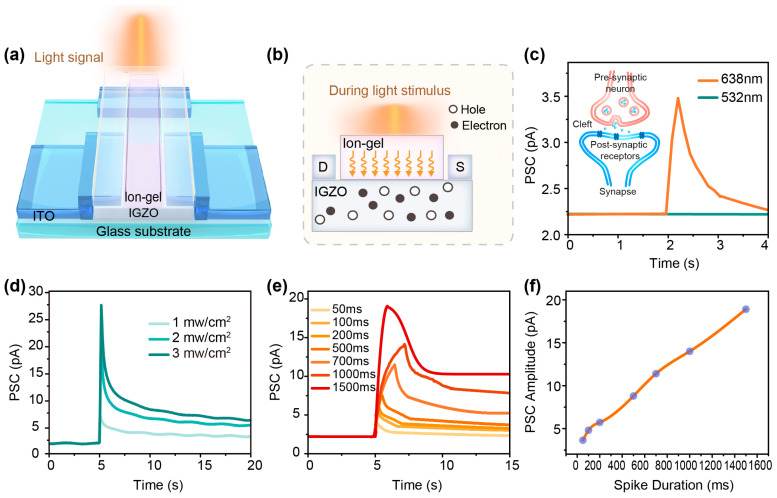
(**a**) A schematic of visible light-modulated synaptic behavior in the a-IGZO phototransistor. (**b**) The carrier generation mechanism under light illumination. (**c**) Transient postsynaptic current (PSC) induced by a 638 nm light pulse (50 ms duration, 1 mW/cm^2^). Inset: Neurotransmission dynamics at biological synapses. (**d**) A comparison of 532 nm light pulse-induced PSC responses under varying optical power densities (1–3 mW/cm^2^). (**e**) PSC behaviors triggered by 638 nm light pulses with increasing duration time (50–1500 ms). (**f**) Light pulse period-dependent absolute PSC amplitude (638 nm, 1 mW/cm^2^).

As shown in Figure 2a, optical stimulation was applied to the transistor channel to emulate neurosynaptic functionality. The underlying mechanism of photogenerated carrier dynamics is schematically illustrated in Figure 2b. When laser irradiation penetrates the optically transparent ion gel layer(post-UV-curved) and interacts with the a-IGZO channel, it generates electron–hole pairs [29]. Due to the presence of persistent photoconductivity (PPC), photogenerated electron–hole pairs do not undergo immediate carrier annihilation after light illumination cease but instead undergo dynamic recombination processes, resulting in a sustained elevation of carrier concentration within the channel. Notably, the temporal evolution of electron generation and recombination processes within a-IGZO under illumination and post-illumination bear remarkable similarity to ion flux dynamics in biological synapses [33], suggesting potential for biomimetic neuromorphic applications.

In biological neural systems, synapses serve as fundamental units for signal transmission between neurons [1]. The inset of Figure 2c schematically illustrates this biological process: When an electrical signal propagates to the presynaptic terminal, it activates ion channels (Na^+^, K^+^, and Ca^2+^ channels) and induces cation influx. This triggers the release of neurotransmitters from synaptic vesicles into the synaptic cleft, which subsequently bind to receptors on the postsynaptic membrane, inducing transient membrane potential changes [34]. In biological systems, this process typically terminates within tens of milliseconds due to rapid ion clearance mechanisms [35]. Significantly, our a-IGZO neuromorphic transistor demonstrates analogous optoelectronic behavior. As shown in Figure 2c, visible light stimulation induces transient channel current responses, resembling biological postsynaptic currents. This light-modulated conductivity mimics the ion-dependent plasticity observed in biological synapses, confirming the device’s potential for neuromorphic computing applications. Herein, our transparent conducting IGZO-based thin-film transistor (TFT) employs visible light laser pulses (450 nm, 532 nm, and 638 nm) as presynaptic stimuli, with the photocurrent through the indium gallium zinc oxide (a-IGZO) channel serving as the postsynaptic response. During operation, light spikes—acting as presynaptic inputs—are incident upon the a-IGZO channel of the neuromorphic transistor under a constant drain voltage, where the drain current and channel conductance are analogous to the postsynaptic current and synaptic weight, respectively. Although the fabricated device cannot replicate the complexity of biological synapses, it effectively mimics key neuromorphic functions.

Figure 2c, which we previously referenced, demonstrates a typical transient postsynaptic current (PSC) elicited by a 638 nm red light pulse (duration: 50 ms; power intensity:1 mW/cm^2^). Under these conditions, a constant V_ds_ = 0.1 V is applied to the ITO drain electrode. The PSC exhibits an immediate increase, reaching a peak absolute amplitude of 3.5 pA upon laser illumination, followed by a gradual decay due to PPC—a phenomenon discussed above when photo-generated charge carriers persist beyond the light pulse duration. Ultimately, the PSC returns to the baseline photocurrent level within several seconds, mirroring the short-term plasticity (STP) observed in biological synapses [36], which is critical for information encoding and transmission [37]. The entire rising phase of the photoresponse demonstrated a response time of approximately 250 ms, underscoring the rapid response capability of IGZO phototransistors even under low-energy red light stimulation. Remarkably, for 532 nm light at the same power density, no optical response is observed unless the pulse width exceeds 150 ms. Within this pulse width range, increasing the optical power density fails to induce a corresponding synaptic response, highlighting the device’s wavelength/time-dependent photoresponsivity. In addition, pronounced light-induced synaptic behaviors are observed for pulse durations exceeding 150 ms at 532 nm, as shown in Figure 2d, which reveals a positive correlation between the absolute PSC amplitude and optical power density for fixed pulse duration.

The experimental results presented above imply that the photoelectric response of IGZO to visible light does not stem from conventional inter-band transition processes. This study proceeded to examine artificial synaptic responses under 638 nm illumination using monophasic light pulses with durations ranging from 50 to 1500 ms. A comparative analysis of the induced PSC processes is presented in Figure 2e. Notably, light pulses exceeding 1000 ms, particularly those surpassing 1500 ms, result in sustained elevated PSC levels that persist beyond the termination of illumination. This phenomenon is attributed to the IGZO channel’s prolonged high-conductance state, maintained through continuous photoconductivity activation during extended exposure periods. Such behavior aligns with the characteristics of long-term synaptic plasticity (LTP) [38], a phenomenon associated with enduring modifications in synaptic strength underlying memory formation and adaptive neural reorganization [39]. Figure 2f illustrates the PSC amplitude as a function of light pulse duration, showing a gradual increase from 3.5 pA to 18.9 pA as the pulse duration extends from 50 ms to 1500 ms. The energy consumption of a single optical synapse event can be quantitatively determined using the formula E= × $V_{ds}$ × $T_{pulse}$, where $I_{PSC}$ denotes the peak postsynaptic current, $V_{ds}$ represents the applied readout voltage (0.1 V), and $T_{pulse}$ corresponds to the duration of the optical pulse. Through this calculation, the device achieves a minimum single-event energy consumption of 17.5 fJ, which aligns closely with the energy scales observed in biological synaptic transmission [40]. The incident energy for this process (50 ms red light pulse) was approximately 98 nJ. These results underscore the significant potential of IGZO-based optoelectronic systems in low-power neuromorphic applications, particularly for energy-efficient neural computation and information processing.

**Figure 3:**
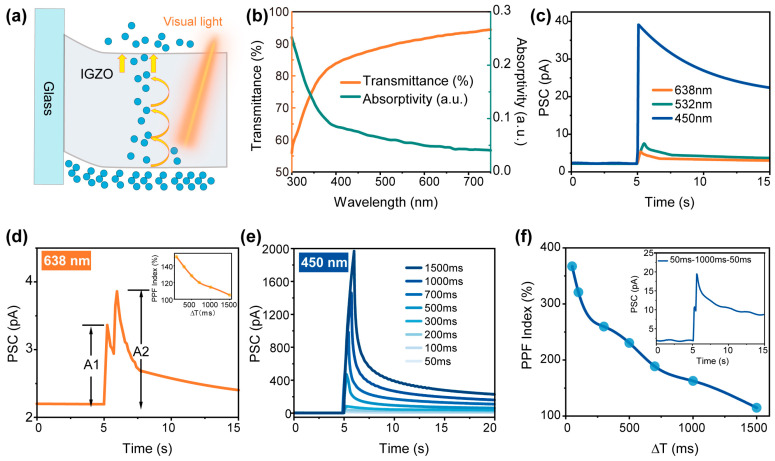
(**a**) The subgap state-mediated photon absorption mechanism: the stepwise excitation of trapped electrons enables a visible light response. (**b**) Optical transmittance and absorbance spectra of the a-IGZO thin film. (**c**) Wavelength-dependent PSC responses (450, 532, and 638 nm; 200 ms pulse duration, 1 mW/cm^2^). (**d**) Paired-pulse facilitation (PPF) behavior induced by dual 638 nm pulses (50 ms duration, ∆T = 1000 ms). Inset: PPF index decay as a function of inter-pulse interval (∆T); (**e**) 450 nm light pulse-induced PSC responses with varying durations (50–1500 ms, 1 mW/cm^2^); (**f**) 450 nm light pulse-induced PPF index decay as a function of ∆T. Inset: PPF behavior induced by dual 450 nm pulses (50 ms duration, ∆T = 1000 ms).

Imperfect IGZO typically exhibits a bandgap of approximately 3.0 eV. Its photoelectric response to visible light in the red spectrum (638 nm) can be attributed to interactions between low-energy photons and subgap states generated during IGZO growth. Subgap states refer to localized energy levels or defect states distributed within the bandgap, distinct from the conduction band minimum or valence band maximum. These states are primarily induced by structural defects, such as oxygen vacancies [41]. When incident photons (e.g., 1.96 eV for 638 nm light) with energy below the bandgap threshold irradiate the IGZO channel, the photons are initially absorbed by these subgap states, which act as intermediate energy levels. Subsequently, the photogenerated electrons are thermally excited to higher energy levels and ultimately injected into the IGZO conduction band, thereby contributing to enhanced photocurrent [42]. This stepwise carrier excitation mechanism is schematically illustrated in Figure 3a [43]. Experimental evidence indicates that the formation of subgap states is highly dependent on growth conditions. Specifically, oxygen vacancies—primary contributors to subgap states—are generated in IGZO films during magnetron sputtering when oxygen supply is insufficient. Increased oxygen vacancy concentrations elevate subgap state density, thereby enhancing the visible light absorption capabilities of IGZO. Furthermore, post-deposition annealing at 150 °C stabilizes defect states and enhances crystallinity, thereby optimizing both optical absorption and electrical performance [29,44]. The absorbance a nd transmittance spectra of the as-grown a-IGZO film on a glass substrate (Figure 3b) reveal differential absorption behavior at 450 nm, 532 nm, and 638 nm, which correlate with the photoelectric response characteristics observed in subsequent transistor measurements (Figure S3). These results are consistent with previous research on the wavelength-dependent photoresponsivity of a-IGZO devices under visible light excitation.

Experimental observations demonstrate that the absorption-excitation mechanism relying on subgap states within the band gap exhibits distinct characteristics compared to the conventional direct interband transition. Typically, as the incident light wavelength decreases, the corresponding photon energy increases, enabling deeper penetration into the band gap. This results in the excitation of a greater number of subgap states located further within the bandgap, facilitating electron transport from these states to the conduction bandand enhancing photocurrent. However, due to the specific energy level distribution of the subgap states in the fabricated device, the rate at which photogenerated electrons reach the conduction band becomes a critical factor in determining the PSC amplitudes for both red (638 nm) and green (532 nm) light under narrow pulse duration. Within a 150 ms time frame, red light with lower photon energy is observed to induce light synaptic behavior more readily. In contrast, a longer pulse duration is required for the induction of arrested PSC current with green light. This phenomenon can be analogized to a multiplet-step transport mechanism, where red light enables a more rapid electron transfer through subgap states to the conduction band, akin to ascending three steps per stride is faster than ascending one step when traversing a staircase with uniformly spaced steps. However, incompatible step sizes (e.g., one and a half steps) result in the slowest electron transport. When the light pulse duration is sufficiently extended to allow for the majority of photogenerated electrons to reach the conduction band, the number of photogenerated carriers begins to dominate the PSC intensity regulation. Subsequent experiments have corroborated this hypothesis, as demonstrated in Figure 3c, where concurrent light synaptic behavior induced by the three wavelengths at identical pulse widths and optical power densities results in an increase in absolute PSC amplitude with decreasing wavelength, consistent with theoretical predictions. Similarly, higher light intensities result in more photons being absorbed by subgap-trapped electrons, which are then excited to the conduction band in a stepwise manner, thereby enhancing the photocurrent.

In the context of the absorption–excitation mechanism relying on subgap states within the band gap, a pair of rarely investigated 638 nm light pulses were selected to model neural facilitation behavior. Paired-pulse facilitation (PPF), a short-term plasticity, is critical for decoding temporal information in biological neural systems, which could be characterized by an increased peak amplitude of the second photo-induced current (A2) compared to the initial response (A1) when two successive stimuli are applied in close temporal proximity. The PPF index is defined by the following equation:

(1) $$PPF\;Index=\frac{A_{2}}{A_{1}}\times 100\%$$

Experimental results demonstrate that applying two consecutive laser pulses (1 mW/cm^2^, 50 ms) with a 1000 ms interval (∆T) on the a-IGZO channel results in that A2 (3.9 pA) exceeds A1 (3.4 pA) by 15%, confirming the successful emulation of PPF in biological synapses. A systematic evaluation of the PPF index curve was conducted using input spikes with controlled interval time. The results reveal an increase in the second PSC amplitude with decreasing ∆T, accompanied by a clear temporal decay of the PPF index as a function of inter-pulse interval, as illustrated in the inset of Figure 3d. Analogous light synaptic behaviors were observed using 450 nm and 532 nm laser pulses.

The significant enhancement in PSC amplitude with spike durations spanning 50–1500 ms, as evidenced by Figure 3e, demonstrates that 450 nm photons (higher energy) induce an increased population of excited electrons. The difference in EPSC amplitude observed between red and blue light in IGZO optoelectronic transistor arises from two factors: (1) the relatively insufficient density of low-energy subgap states, which limits carrier excitation under red light illumination, and (2) the dominance of direct inter-band transitions at higher photon energies, as indirectly evidenced by the PPF behavior triggered by 450 nm dual-pulse excitation. Figure 3f presents the standard PPF response of a 450 nm laser pulse (1 mW/cm^2^, 50 ms, ΔT = 1000 ms), with the corresponding PPF index curve versus ΔT provided for reference. The data reveal a maximum PPF index of 367% at ΔT = 50 ms, significantly exceeding the 151% maximum observed under 638 nm illumination. This discrepancy is attributed to the multi-step absorption–excitation mechanism of subgap states, which involves thermally assisted electron excitation governed by Boltzmann statistics. Consequently, only a fraction of photogenerated electrons from the second red light pulse can be thermally excited to higher energy levels and ultimately injected into the conduction band, resulting in diminished PPF behavior. In contrast, direct inter-band transitions occur in a single-step process without such probability limitations. Synaptic behaviors, including STP, LTP and PPF based on 532 nm light, are further detailed in Figure S4.

**Figure 4:**
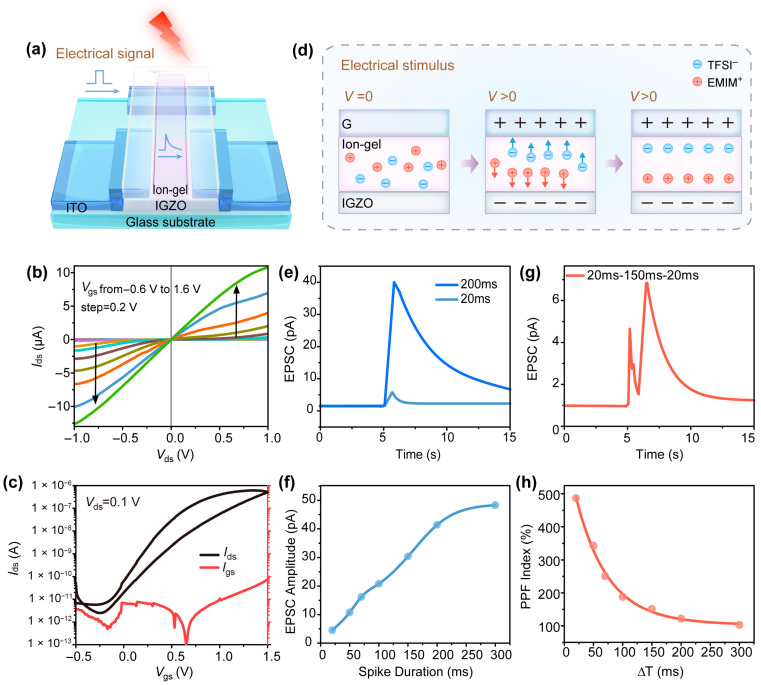
(**a**) A schematic of the electrically modulated TFT transistor via coplanar ion gel gating. (**b**) Output characteristic curves of the transistor. (**c**) The bidirectional transfer characteristic curve of the transistor at V_ds_ = 0.1 V. (**d**) Ion migration dynamics in the ion gel under gate bias: EDL formation modulates channel conductivity. (**e**) Excitatory postsynaptic current (EPSC) triggered by 20 ms and 200 ms electrical pulses (0.1 V amplitude). (**f**) EPSC amplitude dependence on electrical pulse duration (0.1 V, 20–300 ms). (**g**) PPF behavior induced by paired electrical pulses (20 ms duration, ∆T = 150 ms). (**h**) The variation of PPF index with ∆T and its corresponding fitting curve.

The electrical characteristics of the a-IGZO neuromorphic transistor were subsequently investigated, as illustrated in Figure 4a. The device operates under electrical signals applied directly to the coplanar ITO gate electrode. The carrier concentration within the semiconductor channel is modulated through the electric double layer (EDL) effect of the ion gel, which effectively tunes the IGZO channel conductivity. The EDL properties of the ~2-µm-thick ion gel (determined via photoresist notch) were validated through a frequency-dependent phase angle and capacitance analysis, as detailed in Figure S5. Under applied side gate voltage bias, the transistor operates at reduced gate voltages (≤2.0 V) and low source-drain voltages, thereby minimizing power consumption. The output characteristic curves, shown in Figure 4b, were obtained by scanning the drain-source voltage (V_ds_) from −1 V to 1 V while varying the side gate voltage (V_gs_) from −0.6 V to 1.6 V in 0.2 V increments. The Ids curve exhibits linear behavior in the low-voltage region and saturation characteristics at a higher voltage, consistent with typical n-channel transistor behavior with ohmic contact. The transfer characteristic curve, a critical determinant of transistor performance, is presented in Figure 4c with a fixed V_ds_ of 0.1 V. A switching ratio of ~10^5^ is achieved within the scanned voltage range of −0.5 V to 1.5 V, meeting the required switching performance criteria. Additionally, the gate leakage current remains sufficiently low (~10^−12^ A). The transfer curves in Figure 4c demonstrate excellent performance, indicating the significant modulating effect of the ion gel (via subsequent ink-jet printing) on the IGZO channel, primarily attributed to the previously described EDL effect. Key parameters obtained include a low threshold voltage (Vth = 0.17 V), subthreshold swing (SS = 130 mV/decade), and field-effect mobility (μFE = 1.2 cm^2^/(V∙s)). The findings indicate that IGZO semiconductors processed at low temperatures demonstrate exceptional electrical properties, making them highly suitable for future applications on flexible substrates that cannot tolerate high temperatures.

Noteworthy is the observation of a ~0.2 V hysteresis window in the transfer characteristics (Figure 4c) during gate voltage sweep. This phenomenon originates from the delayed ionic migration dynamics within the gel under reverse voltage scanning relative to the electric field response, thereby enabling its application in neuromorphic behavior simulation through transient charge storage mechanisms. As previously discussed, in biological neural systems, the arrival of a presynaptic bias input at the axon terminal triggers neurotransmitter release into the synaptic cleft, involving ions such as Na^+^ and Ca^2+^. This, in turn, generates a postsynaptic electrical signal. The ion-gel-coupled transistor could be regarded as an artificial synapse, comprising a presynaptic terminal (ITO side gate electrode) and a postsynaptic terminal (a-IGZO channel). The application of an electrical spike to the ITO gate electrode represents synaptic input strength, inducing an ionic excitatory postsynaptic current (EPSC). The channel conductance is defined as the synaptic weight. Mobile EMIM^+^ TFSI^−^ ions in the ion gel between the gate and channel play a pivotal role in EPSC generation.

Ionic migration dynamics within the gel under electrical stimulation are demonstrated in Figure 4d. Due to the electronically insulating yet ionically conductive nature of ion gels, anions and cations exhibit random distribution within the gel at initial V = 0 V. Consequently, the IGZO channel current under source-drain readout bias remains minimal. Upon applying a presynaptic bias (V > 0), cations within the ion gel undergo rapid electrophoretic migration and accumulate at the ion-gel/IGZO interface under the applied electric field. A corresponding electron layer within the IGZO channel is induced by the ionic polarization effect, thus establishing an electrical double-layer (EDL) structure with submillisecond temporal resolution, while anions exhibit reverse migration trajectories compared to cations during this process. In contrast to optical excitation, where photon absorption in IGZO directly generates photoexcited carriers to elevate channel carrier concentration, electrical pulse stimulation modulates carrier concentration through the capacitive effect of transient electric double-layer (EDL) structures formed at the electrolyte/semiconductor interface. Prolonged electrical stimulation facilitates greater ion migration to form the EDL structure, amplifying its capacitive contribution, which subsequently enhances the channel current under readout voltages owing to increased mobile carrier availability. The ionic species constituting the electric double-layer (EDL) structure in ion gels predominantly accumulate at the gel/IGZO interface under electrostatic bias, exhibiting negligible transinterface migration. Consequently, upon termination of the applied electrical bias, these ions undergo reversible recovery to their initial equilibrium states. The ion migration hysteresis observed in the transfer curve’s hysteresis window dictates that this recovery process does not occur instantaneously with bias removal but instead proceeds through dynamic relaxation mechanism. EPSC current in undoped IGZO channels demonstrates progressive decay during the ionic relaxation phase, with the current magnitude inversely correlating with the degree of ion re-equilibration at the interface.

During neuromorphic function testing, a constant V_ds_ = 0.05 V and pulse amplitude of 0.1 V were applied. Figure 4e demonstrates two representative EPSC synaptic responses in the IGZO-based EDL transistor. The EPSC amplitude reaches only a few picoamperes (pA) during pulse durations of tens of milliseconds, followed by rapid decay within seconds due to the reverse diffusion of EMIM^+^-TFSI^−^ ions, effectively mimicking the temporal dynamics of biological EPSCs. This small EPSC enhancement coupled with rapid decay is characterized as short-term plasticity (STP). As the pulse duration increases, both the EPSC amplitude and ion decay time exhibit progressive enhancement, attributed to the trapping and accumulation of more cations at the semiconductor interface under prolonged electrical stimulation. The response of a-IGZO synaptic transistor shown in Figure 4e to 200 ms presynaptic voltage spike with a long decay time (tens of seconds) and elevated EPSC magnitude is a hallmark feature of long-term potentiation (LTP).The pulse-duration-dependent EPSC amplitude characteristics in the synaptic transistor are demonstrated in Figure 4f, exhibiting an initial linear increase in EPSC magnitude with prolonged presynaptic pulse durations. However, when the spike duration exceeds 250 ms, the capacitance-induced carrier accumulation reaches saturation, leading to stabilized EPSC amplitudes. This nonlinear behavior is attributed to the interfacial charge storage mechanism, which arises from the limited mobile ion concentration within the electrolyte layer and the formation of a stable EDL structure under extended bias conditions. The stabilized EDL configuration exhibits spatial scalability with the contact area between the ion gel and ITO side-gate electrodes, enabling tunable charge storage capacity through geometric modulation of the device architecture. Furthermore, the electrical energy consumption per EPSC demonstrates a functional dependence on the duration of the electrical spike, where the energy of the per-synaptic event ranges from a minimum of 4.5 fJ (20 ms, 0.1 V pulse, V_ds_ = 0.05 V) to a maximum of 735 fJ [45]. This energy consumption level exhibits close comparability to biological synaptic operations while aligning with other ultra-low-power ionic gel-based artificial synapses in the same order of magnitude, thereby highlighting the device’s potential for energy-efficient synaptic application in an ion-gel neuromorphic system [45,46].

It is noteworthy that despite the ion-conductive yet electronically insulating nature of the ion gel, experimental observations revealed that when the gate bias exceeded 300 mV or pulse durations surpassed 350 ms during testing, the ion gel exhibited irreversible breakdown phenomena. This breakdown induced uncontrolled electrochemical doping processes, where mobile ions penetrated the interface and migrated into the a-IGZO channel, hindering electrical measurements. The maximum doping-voltage/pulse-duration threshold is related to the EMIM-TFSI weight percentage in the ionic liquid mixture. However, as this ratio decreases, EDL capacitance diminishes [47,48], weakening the ion gel’s regulation of channel conductivity. Therefore, the proportion must be meticulously selected to ensure optimal performance and reliability. Furthermore, ITO electrodes are prone to faradaic reactions when in contact with an ion gel [49], requiring precise control of oxygen/argon gas ratios during ITO film deposition to balance conductivity and transparency.

Paired electrical stimuli were applied to the IGZO neuromorphic transistor to emulate paired-pulse facilitation (PPF). Two presynaptic spikes (0.1 V, 20 ms) with a 150 ms interval (∆T) and constant V_ds_ = 0.05 V were used to elicit EPSC, as shown in Figure 4g. The results reveal that the second EPSC amplitude (A2) is 128% higher than the first, demonstrating typical PPF behavior. This phenomenon can be attributed to ionic relaxation dynamics: ions triggered by the initial pulse have insufficient time to diffuse back into the gel if the subsequent pulse occurs within a short interval, and they participate in the formation of a new EDL structure, thereby strengthening the second EPSC. The A2/A1 ratio versus ∆T is shown in Figure 4h, reaching a maximum of 480% at ∆T = 20 ms and decreasing to 105% as ∆T increases from 20 ms to 300 ms. However, no facilitation was observed for pulse intervals exceeding 350 ms, indicating complete ionic equilibration. The PPF index could be quantitatively characterized via a fitting curve of the experimental data shown in Figure 4h. The fitting formula is mathematically expressed as follows [46]:

(2) $$PPF\;Index=C_{1}exp\left(-\frac{\Delta T}{\tau_{1}}\right)+C_{2}exp\left(-\frac{\Delta T}{\tau_{2}}\right)$$

where ∆T denotes inter-pulse interval between two successive electrical stimuli; $C_{1}$ and $C_{2}$ represent the initial facilitation degrees of the fast and slow relaxation components, respectively; and $\tau_{1}$ and $\tau_{2}$ correspond to the characteristic relaxation times of these two phases. Curve fitting yielded relaxation times of $\tau_{1}$ = 53 ms and $\tau_{2}$ = 167 ms, which align with the temporal scales reported for paired-pulse facilitation in biological synapses [50], which validates the feasibility of simulating synaptic behaviors using ink-jet-printed ion gels within transistor architectures.

## 3. Conclusions

This work demonstrates a scalable and low-temperature-fabricated ion gel-gated a-IGZO neuromorphic transistor with multispectral responsivity. The device successfully emulates key neurosynaptic behaviors, including short-term plasticity (STP), long-term plasticity (LTP), and paired-pulse facilitation (PPF), using both optical and electrical stimuli. The implementation of ink-jet printing technology and low-temperature processes ensures compatibility with flexible substrates and industrial-scale production. This platform provides a promising route toward multifunctional AI ocular neural systems, enabling efficient and low-power synaptic computation in vision-related applications. However, to expand the applicability of devices in practical neuromorphic systems, further optimization of the mass ratios of ionic gel components are required to achieve a synergistic combination of high capacitance with electrical-breakdown resistance.

## 4. Materials and Methods

Materials: Electronic-grade borosilicate glass substrates (35 × 35 mm^2^) were utilized. Sputtering targets included amorphous In-Ga-Zn-O (a-IGZO) target (99.99%, In_2_O_3_:Ga_2_O_3_:ZnO = 1:1:1, 3-inch diameter), indium–tin–oxide (ITO) target (90 wt% In_2_O_3_, 10 wt% SnO_2_). Photoresists (AZ 601, ROL 7133) and ionic gel precursors were prepared for the experiment: 1-ethyl-3-methylimidazolium bis (trifluoromethyl sulfonyl) imide ([EMIM] [TFSI]), Poly (ethylene glycol) diacrylate (PEGDA), 2-hydroxy-2-methylpropiophenone (HOMPP).

Ion Gel Preparation: 1-Ethyl-3-methylimidazolium bis (trifluoromethyl sulfonyl) imide (EMIM-TFSI), poly (ethylene glycol) diacrylate (PEGDA), and 2-hydroxy-2-methyl-propiophenone (HOMPP) were mixed with a quality ratio of 87:9:4. The mixed liquid was magnetically stirred for 5 h in a dark environment at room temperature. The mixture was vacuum-dried (70 °C, 8 h, <10^−3^ Torr) to residual moisture. The dehydrated precursor was stored at 4 °C in amber vials before ink-jet deposition.

Substrate Preparation: Substrates underwent sequential ultrasonic cleaning (15 min each) in acetone, ethanol, and deionized water, followed by N_2_ blow-drying. A 40 nm a-IGZO layer was deposited via RF magnetron sputtering (150 °C, 3.7 × 10^−3^ Torr, Ar_2_ atmosphere). Post-deposition annealing (150 °C, 60 min, N_2_) enhanced stoichiometric uniformity. The layer was patterned by photolithography (AZ 601) and wet-etched in 2 vol% HCl. ITO electrodes (source/drain/gate) that were 100 nm thick were sputter-deposited (RT, 6 × 10^−3^ Torr) and patterned via lift-off processing (ROL 7133).

Ion Gel Integration: AZ 601 photoresist was spin-coated (1500 rpm, 60 s) onto the prepared device to be patterned to define ion gel regions (1100 × 90 μm^2^). The precursor was ink-jet-printed (SIJ S050, SIJ Technology, Tokyo, Japan) and UV-cured in two-stage illumination (365 nm, 30 s × 2 cycles).

Device characterization: The surface properties of IGZO were measured through atomic force microscopy (AFM). The optical absorbance and transmissivity spectra of a-IGZO layer grown on the glass substrate were identified by an ultraviolet–visible spectrometer. The capacitance characteristics of the ion gel were measured by an impedance analyzer. The optoelectronic properties and neuromorphic behavior of the a-IGZO transistors were evaluated using a 4200 SCS (Keithley, Beaverton, Oregon, United States) semiconductor parameter analyzer in a dark environment at room temperature in ambient air. Several lasers with representative visible wavelengths of 450 nm (blue), 532 nm (green), and 638 nm (red) were prepared as sources of visible input peaks.

## Data Availability

The original contributions presented in this study are included in the article. Further inquiries can be directed to the corresponding authors.

## Supplementary Materials

The following supporting information can be downloaded at: https://www.mdpi.com/article/10.3390/s25092750/s1, Figure S1: An AFM topography image (a) and 3D schematic illustration (b) of the IGZO film surface. Figure S2: A schematic diagram of the ink-jet printing process for fabricating ion gel. Figure S3: The photocurrent response of the phototransistor under the illumination of visible light with three different wavelengths (405 nm, 532 nm, and 638 nm) in the absence of an ion gel. Figure S4. (a) PSC behaviors triggered by 532 nm light pulses with increasing duration time. (b) Light pulse period-dependent absolute PSC amplitude (150–1500 ms, 1 mW/cm^2^). (c) Paired-pulse facilitation (PPF) behavior induced by dual 532nm pulses (150 ms duration, ∆T = 700 ms). (d) PPF index decay as a function of the inter-pulse interval (∆T). Figure S5. The frequency dependence of capacitance (μF/cm^2^) and phase angle (°) of the ion gel.
