# Supplementary material for: Ion Gel-Modulated Low-Temperature Field-Effect Phototransistors with Multispectral Responsivity for Artificial Synapses
Source: Sensors (Basel). 2025 Apr 26;25(9):2750. doi: 10.3390/s25092750 (PMC12074399; doi:10.3390/s25092750)
Supplement: Supplementary file 1 [file sensors-25-02750-s001.zip › sensors-3577681-supplementary.pdf]

Supporting Information

Ion Gel-Modulated Low-Temperature Field-Effect Phototransistors with Multispectral Responsivity for Artificial Synapses

Junjian Zhao [1,2], Yufei Zhang [1,2], Di Guo [1,2,*] and Junyi Zhai [1,2,3,*]

[1] Beijing Key Laboratory of Micro-Nano Energy and Sensor, Center for High-Entropy Energy and Systems, Beijing Institute of Nanoenergy and Nanosystems, Chinese Academy of Sciences, Beijing 101400, China; zhaojunjian@binn.cas.cn (J.Z.); zhangyufei@binn.cas.cn (Y.Z.)
[2] School of Nanoscience and Engineering, University of Chinese Academy of Sciences, Beijing 100049, China
[3] Center on Nanoenergy Research, School of Physical Science & Technology, Guangxi University, Nanning 530004, China
* Correspondence: guodi@binn.cas.cn (D.G.); jyzhai@binn.cas.cn (J.Z.)

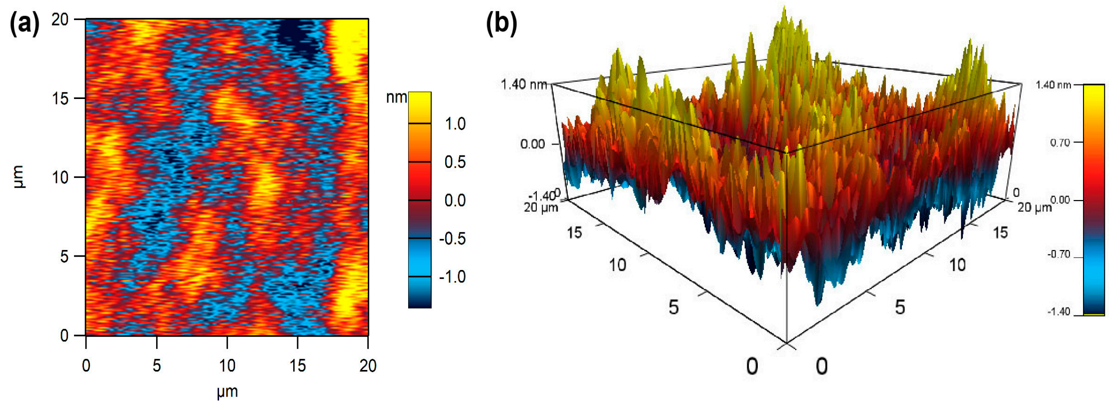

Figure S1. An AFM topography image (a) and 3D schematic illustration (b) of the IGZO film surface.

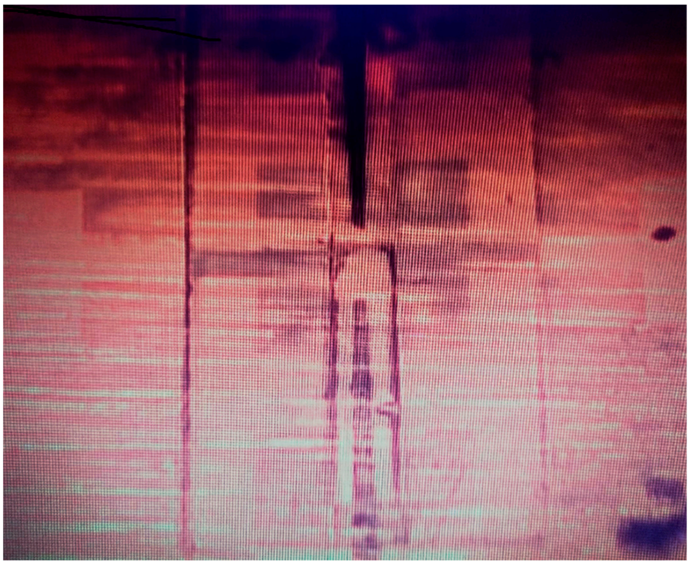

Figure S2. A schematic diagram of the ink-jet printing process for fabricating ion gel.

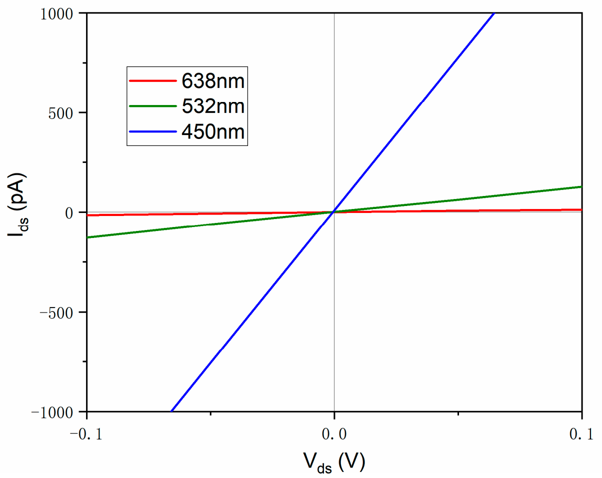

Figure S3. The photocurrent response of the phototransistor under the illumination of

visible light with three different wavelengths (405 nm, 532 nm, and 638 nm) in the absence of ion gel.

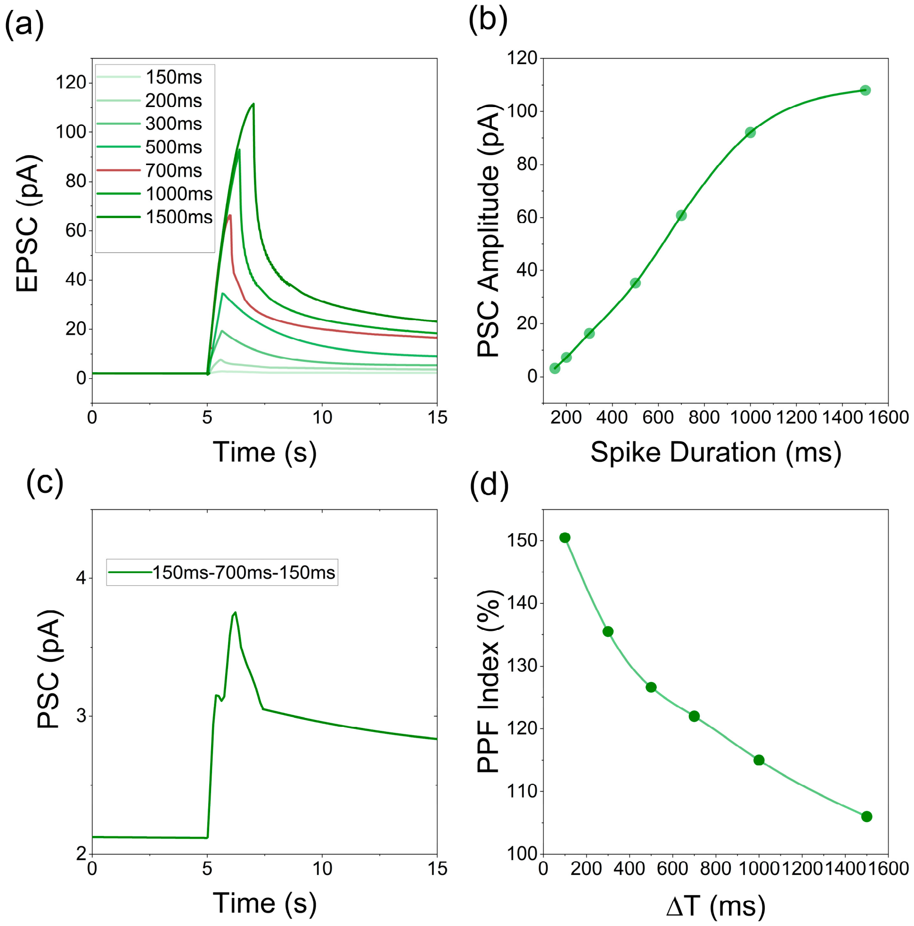

Figure S4. (a) PSC behaviors triggered by 532 nm light pulses with increasing duration time. (b) Light pulse period-dependent absolute PSC amplitude, (150–1500 ms ,1 mW/cm²). (c) Paired-pulse facilitation (PPF)behavior induced by dual 532nm pulses (150ms duration, ΔT=700ms). (d) PPF index decay as a function of the inter-pulse interval(ΔT).

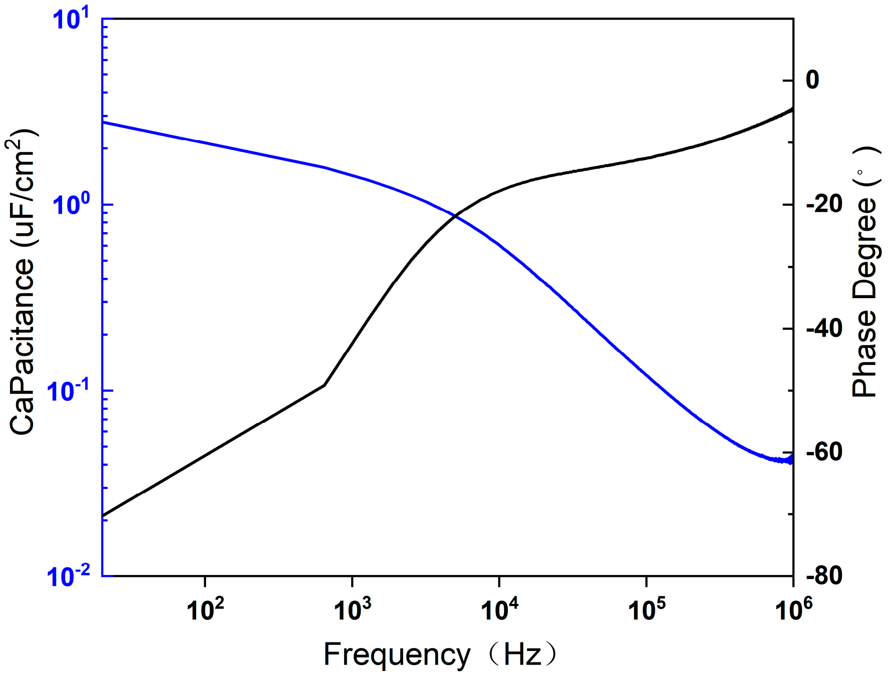

Figure S5. The frequency dependence of capacitance (μF/cm²) and phase angle (°) of the ion gel.
